# Supplementary material for: The genome sequence of the Broad-barred Knot-horn, Acrobasis consociella (Hübner, 1813)
Source: Wellcome Open Res. 2024 Aug 7;9:429. [Version 1] doi: 10.12688/wellcomeopenres.21553.1 (PMC11624432; doi:10.12688/wellcomeopenres.21553.1)
Supplement: Peer review history

# Reviewer report: Reviewer response for version 1

**DOI:** 10.21956/wellcomeopenres.23824.r112675

**Published:** 2024-12-06

**Authors:** Jerome H L Hui

**Affiliations:** 1 The Chinese University of Hong Kong, Hong Kong, Hong Kong

In this data note, Boyes and colleagues reported the genome sequence of a female moth *Acrobasis consociella *(Hübner, 1813). According to the UKMoth, *A. consociella *is a "reasonably common species distributed from southern England northwards into southern Scotland and west into Ireland". Prior to this report, molecular data available for this species are rather limited (which are mainly mitochondrial cytochrome oxidase subunit 1 (COI) gene sequences deposited to the NCBI database). The newly generated genomic resource of this species is important and useful for further studies, including but not limited to understanding the genomics, ecology, impact of climate change, insect-plant interaction, identification of cryptic species, reveal of population structure, to understanding their evolution with other insects.

 This genome resource is excellent from the summary statistics, with high BUSCO number scores, high sequence continuity (scaffold N50), and majority of sequences contained on the 29 pseudochromosomes (plus sex chromosomes and mitochondrion). All in all, this is a valuable contribution.

Are sufficient details of methods and materials provided to allow replication by others?

Yes

Is the rationale for creating the dataset(s) clearly described?

Yes

Are the datasets clearly presented in a useable and accessible format?

Yes

Are the protocols appropriate and is the work technically sound?

Yes

Reviewer Expertise:

Genomics, evolution, invertebrates

I confirm that I have read this submission and believe that I have an appropriate level of expertise to confirm that it is of an acceptable scientific standard.

# Reviewer report: Reviewer response for version 1

**DOI:** 10.21956/wellcomeopenres.23824.r104475

**Published:** 2024-10-29

**Authors:** Frederico Hickmann

**Affiliations:** 1 The Ohio State University College of Food Agricultural and Environmental Sciences (Ringgold ID: 155699), Wooster, Ohio, USA

This manuscript provides a comprehensive account of the complete genome sequencing, assembly, and annotation of *Acrobasis consociella*, a species of the Pyralidae family indigenous to Great Britain. The work presented is meticulous and well-structured, employing appropriate genome assembly tools relevant to current and forthcoming research endeavors. The assembly corresponds with the standard chromosome count typically observed in Pyralidae moths. Moreover, the initiative to disseminate this data openly is of considerable importance to the scientific community.

Are sufficient details of methods and materials provided to allow replication by others?

Yes

Is the rationale for creating the dataset(s) clearly described?

Yes

Are the datasets clearly presented in a useable and accessible format?

Yes

Are the protocols appropriate and is the work technically sound?

Yes

Reviewer Expertise:

My area is agricultural entomology, focusing on molecular ecology, genomics, and pests' off-season persistence.

I confirm that I have read this submission and believe that I have an appropriate level of expertise to confirm that it is of an acceptable scientific standard.
